# Dietary calcium affects body composition and lipid metabolism in rats

**DOI:** 10.1371/journal.pone.0210760

**Published:** 2019-01-10

**Authors:** Haya Alomaim, Philip Griffin, Eleonora Swist, Louise J. Plouffe, Michelle Vandeloo, Isabelle Demonty, Ashok Kumar, Jesse Bertinato

**Affiliations:** 1 Nutrition Research Division, Bureau of Nutritional Sciences, Health Products and Food Branch, Health Canada, Ottawa, Ontario, Canada; 2 Department of Biochemistry, Microbiology and Immunology, University of Ottawa, Ottawa, Ontario, Canada; 3 King Saud bin Abdulaziz University for Health Sciences, Al Hars Al Watani, Ar Rimayah, Riyadh, Saudi Arabia; University of Illinois, UNITED STATES

## Abstract

Calcium (Ca) intakes may affect cardiovascular disease risk by altering body composition (body weight and fat) and serum lipid profile, but results have been inconsistent and the underlying mechanisms are not well understood. The effects of dietary Ca on body composition and lipid metabolism were examined in rats. Male Sprague-Dawley rats were fed high-fat, high-energy diets containing (g/kg) low (0.75Ca, 0.86 ± 0.05; 2Ca, 2.26 ± 0.02), normal (5Ca, 5.55 ± 0.08) or high (10Ca, 11.03 ± 0.17; 20Ca, 21.79 ± 0.15) Ca for 10 weeks. Rats fed the lowest Ca diet (0.75Ca) had lower (*p* < 0.05) body weight and fat mass compared to other groups. Rats fed the high Ca diets had lower serum total and LDL cholesterol compared to rats fed normal or low Ca. Liver total cholesterol was lower in rats fed high compared to low Ca. In general, liver mRNA expression of genes involved in cholesterol uptake from the circulation (*Ldlr*), cholesterol synthesis (*Hmgcr* and *Hmgcs1*), fatty acid oxidation (*Cpt2*) and cholesterol esterification (*Acat2*) were higher in rats fed higher Ca. Apparent digestibility of total trans, saturated, monounsaturated and polyunsaturated fatty acids was lower in rats fed the high compared to the low Ca diets, with the largest effects seen on trans and saturated fatty acids. Fecal excretion of cholesterol and total bile acids was highest in rats fed the highest Ca diet (20Ca). The results suggest little effect of dietary Ca on body composition unless Ca intakes are very low. Decreased bile acid reabsorption and reduced absorption of neutral sterols and saturated and trans fatty acids may contribute to the better serum lipid profile in rats fed higher Ca.

## Introduction

Calcium (Ca) is a mineral nutrient rich in milk products and plays an important role as a second messenger and in signal transduction [1]. Ca is essential for a vast number of physiological processes including bone metabolism, muscle function, nerve transmission, blood coagulation and hormone secretion. Serum ionized Ca concentrations are maintained within a narrow range through the concerted action of parathyroid hormone (PTH), 1,25-dihydroxyvitamin D and calcitonin that act at the intestine, kidneys and bone [2, 3].

Cardiovascular diseases (CVD) are the leading cause of death worldwide [4]. The effects of Ca intakes on CVD risk is currently a topic of debate [5]. Some research suggests that higher Ca intakes may lower body weight or body fat and improve serum lipid-lipoprotein profile and therefore reduce CVD risk [6–11]. However, other research suggests increased risk of cardiovascular events with higher intakes, especially from Ca supplementation [12–14].

Ca intakes may affect body weight and body fat [11, 15–23], but the subject remains contentious given null results reported in some studies [24–28]. Studies in rats and mice have also produced mixed results. Some studies have reported lower body weight and body fat mass with higher Ca intakes [29–32], while other studies have not [33–36]. Decreased appetite, dietary fat absorption and lipogenesis and increased lipolysis and thermogenesis have been proposed as mechanisms to explain the anti-obesity effect of higher Ca intakes [7, 8, 10, 11, 23, 37].

Elevated serum low-density lipoprotein cholesterol (LDL-C) concentrations and low serum high-density lipoprotein cholesterol (HDL-C) concentrations are well-established risk factors for CVD [38]. Higher Ca intakes in rats [29, 39, 40] and other animals [41–43] have been associated with a change in serum lipid profile predictive of a lower risk for CVD. Human studies have also shown improved serum lipid-lipoprotein profile with higher Ca intake [44–47], but not all studies have confirmed these findings [48–50].

The interactions of Ca with lipids and/or bile acids in the gastrointestinal tract (GI) may explain the favourable effect of higher Ca intakes on blood lipids. Ca or Ca-phosphate complexes may increase fecal excretion of bile acids causing an increased demand for hepatic cholesterol for the regeneration of bile acids, ultimately depleting hepatic cholesterol [39, 41, 45]. A decrease in hepatic cholesterol may trigger an upregulation of the LDL receptor increasing uptake of LDL from the circulation and lowering serum cholesterol concentrations. Higher Ca intakes may also reduce intestinal cholesterol absorption. An *in vitro* study has suggested that Ca precipitates fatty acids and in turn decreases the cholesterol solubilisation capacity of dietary mixed micelles involved in cholesterol absorption [51]. A study in hamsters has suggested that higher Ca intakes may decrease cholesterol absorption by affecting expression of intestinal cholesterol transporters including ATP binding cassette transporters (ABCG5/8), Niemann-Pick C1-Like 1 (NPC1L1) and microsomal triacylglycerol transport protein (MTP) [41].

Replacing saturated fatty acids (SFA) or trans fatty acids (TFA) in the diet with monounsaturated fatty acids (MUFA) or polyunsaturated fatty acids (PUFA) decreases serum total cholesterol (TC), LDL-C and triglycerides [52, 53]. TFA have an even more detrimental effect on blood lipids than SFA because TFA increase TC:HDL-C ratio and LDL-C compared to SFA [52]. Ca can form insoluble Ca-fatty acid soaps in the GI tract decreasing the absorption of fatty acids. Some, but not all research suggests that SFA are precipitated to a greater extent by Ca compared to MUFA or PUFA [51, 54]. To date, there is little information on the effect of Ca on TFA absorption. It is conceivable that a greater reduction in absorption of SFA or TFA by Ca compared to MUFA or PUFA may have a favourable effect on serum lipid profile.

Ca intakes may modify CVD risk by affecting body composition or serum lipids, but results to date have been mixed. Thus, this study examined the effects of dietary Ca above and below nutrient requirements on body composition and serum lipids in rats fed a high-fat, high-energy diet. To gain insight into the possible underlying mechanisms driving Ca-dependent changes in these CVD risk factors, energy intake, fatty acid digestibility, fecal excretion of neutral sterols and bile acids and lipid metabolism in the liver were examined.

## Materials and methods

### Diets and animal protocol

One hundred and fifty male Sprague-Dawley CD rats (Charles River Canada, St. Constant, QC, Canada) at 42 days of age were assigned to 1 of 5 high-fat, high-energy diets (Dyets, Inc., Bethlehem, PA, USA) containing different amounts of Ca (0.75Ca, 0.86 ± 0.05; 2Ca, 2.26 ± 0.02; 5Ca, 5.55 ± 0.08; 10Ca, 11.03 ± 0.17; 20Ca, 21.79 ± 0.15 g Ca/kg diet). Rats were assigned to diet groups (n = 30/diet group) based on initial body weight to ensure that mean body weights were similar for each diet group at the start of the study. Compositions and energy densities of the diets are shown in S1 Table. Diets were formulated using the AIN-93G mineral mix [55] without Ca. Ca was added to the diets as Ca carbonate. Diets were adapted versions of the Research Diets, Inc. D12266B diet used previously to induce obesity in rats [56, 57]. Diets were pelleted for more accurate measurement of food consumption.

Rats were housed singly in solid-bottom cages with a wire grill insert and held in vented racks. Rats were put on a 12:12-h light-dark cycle and had free access to food and demineralised water throughout the study. Food consumption and body weight were measured weekly. Body composition was measured using magnetic resonance imaging (EchoMRI-4in1 system, EchoMRI, Houston, TX, USA) at the beginning (Day 0), middle (Day 35) and end (Day 70) of the study. Feces were collected daily for 7 consecutive days during weeks 3 and 8 of the study. Feces were immediately frozen each day after collection. Feces were freeze-dried and weighed prior to analysis.

After 10 weeks of feeding the diets rats were fasted overnight (12 h) in metabolic cages for collection of urine and then killed the following morning by exsanguination under general isoflurane anesthesia. Blood was collected from the abdominal aorta by syringe and dispensed into blood tubes for isolation of serum (Trace Element Serum tube, Thermo Fisher Scientific, Ottawa, ON, Canada) and plasma (BD vacutainer K_2_EDTA, Thermo Fisher Scientific). Liver was dissected out, weighed and a section of the left lateral lobe was removed and snap-frozen in liquid nitrogen for gene expression experiments. The rest of the liver was frozen on dry ice. Inguinal, retroperitoneal plus perirenal, mesenteric and epididymal adipose depots were dissected out and weighed. Tissues, feces, urine and serum/plasma samples were stored at ‐80°C until analysis. During the study 1 rat in the 0.75Ca group and 2 rats in the 2Ca group died unexpectedly without prior symptoms. Rats were examined post-mortem by a pathologist and the final judgement was sudden death of undetermined cause. Results from these rats are not reported. The Health Products and Food Branch Animal Care Committee of Health Canada approved the experimental protocol (Protocol No.: 2015–010).

### Mineral analyses

Diet samples (~0.5 g) were weighed in quartz beakers and dried overnight in an Isotemp oven (Thermo Fisher Scientific) at 100°C. Samples were ashed using a combination of dry ashing using a Thermo Scientific Lindberg/Blue MTM box furnace (Thermo Fisher Scientific) and wet ashing using concentrated trace metal grade nitric acid (Thermo Fisher Scientific). Ashes were solubilized in dilute nitric acid. Solubilized ashes and urine samples were analyzed for mineral concentrations using a radial view 700 Series inductively coupled plasma optical emission spectrometer (Agilent Technologies Canada Inc., Mississauga, ON, Canada). Operating conditions have been described previously [58]. Standard calibration curves were prepared using the CALEDON-88 multi-element standard (Inorganic Ventures, Christiansburg, VA, USA) and analytical precision was verified using National Institute of Standards and Technology traceable reference material (SCP Science, Baie D’Urfé, QC, Canada).

### Assays

Plasma insulin and glucose concentrations were measure using the Rat Ultrasensitive Insulin ELISA (80-INSRTU-E01, Alpco Diagnostics, Salem, NH, USA) and Glucose Colorimetric Assay Kit (10009582, Cayman Chemical, Ann Arbor, MI, USA), respectively. Plasma PTH concentration was measured using the Rat BioActive Intact PTH ELISA Kit (60–2700, Immutopics, Inc., San Clemente, CA, USA). Serum lipids and minerals and urine creatinine were measured using the ABX Pentra 400 chemistry analyser (HORIBA Instruments Inc., Irvine, CA, USA).

### Measurement of total lipids and fatty acids in feces and diets

Total lipids were extracted from ground, freeze-dried feces and diet samples using an improved Bligh and Dyer extraction procedure [59] with modifications. Briefly, ~100 mg of sample (weighed accurately) was hydrolyzed with 1 mL of 3 M HCL. A 2:1 (v/v) mixture of methanol:chloroform (2.25 mL) and 0.25 mL (0.512 mg) of 13:0 fatty acid internal standard (Nu-Check Prep, Waterville, MN, USA) was added to the samples. Then, 2 mL of chloroform and 1 mL of water was added, the samples were vortexed, centrifuged (5 min, 2000 × g) and the bottom chloroform layer was collected into a pre-weighted glass tube. The chloroform was evaporated under nitrogen and the tube reweighed to obtain the weight of total lipids.

For determination of fatty acid concentrations, total lipids were dissolved in 1 mL of toluene and converted into fatty acid methyl esters (FAME) by adding 0.5 mL of methanol and 0.5 mL of boron trifluoride (in 14% methanol) and heating for 1 h at 105°C. FAME were recovered by adding 2 mL of water and 2 mL of hexane to the sample, mixing and collecting the hexane layer. The hexane was dried with anhydrous sodium sulphate for a minimum of 20 min and the hexane was then transferred to a new tube and evaporated under nitrogen. The FAME were re-solubilised in 1 mL of hexane and 1 ul was analyzed by gas chromatography (Agilent 6890N system with an auto injector; Agilent, Santa Clara, CA, USA). The gas chromatograph was fitted with a flame ionization detector and a 100-m x 0.25-mm capillary column (SP-2560, Sigma Aldrich, Oakville, ON, Canada). The initial column oven temperature was 180°C, followed by 2 ramps at 32 min (to 215°C) and 65 min (to 240°C). The injector and the detector temperatures were 250°C. Ultra-high-purity hydrogen was used as the carrier gas at a flow rate of 0.8 mL/min. Chromatographic peaks were identified by comparison with known FAME standards (Nu-Chek Prep, Waterville, MN, USA and Sigma Aldrich). Concentrations of fatty acids were determined by comparison to the internal standard. Fatty acids representing less than 0.05% of total fatty acids were excluded from further analysis.

### Measurement of lipids in liver

Total lipids were extracted from a large portion of the liver comprising all 4 lobes missing a small section of the left lateral lobe that was used for other analyses. The liver was ground with a tissue grinder into a homogenous slurry and ~300 mg of slurry (weighed accurately) was homogenized in 5 mL of chloroform:methanol (2:1, v/v). The samples were incubated overnight on a shaker, centrifuged (10 min, 2000 × g) and the supernatant was collected. Sodium chloride (0.9%, w/v) was added to the supernatant and the organic solvent layer was recovered and evaporated under nitrogen to obtain the weight of total lipids. For determination of TC, free cholesterol and triglyceride concentrations, total lipids were extracted from an ~300 mg section (weighed accurately) of the left lateral lobe as described above. Total lipids were weighed and re-suspended in 1.5 mL of 10% Triton X-100 in isopropanol. TC, free cholesterol and triglycerides were measured using Wako Cholesterol E (999–02601, Wako Chemicals, Richmond, VA, USA), Free Cholesterol E (993–02501, Wako Chemicals) and L-Type Triglyceride M (Wako Chemicals) kits, respectively.

### Measurement of neutral sterols and bile acids

Neutral sterols and bile acids were analysed following previously published methods [60, 61] with some modifications. Approximately 100 mg of freeze-dried feces or diet (for cholesterol measurement) were weighed accurately and 3 mL of 1 N NaOH (in 90% ethanol) was added to the sample. Then, 0.25 mL of 5α-cholestane (0.110 mg) (Sigma Aldrich) and hyodeoxycholic acid (0.155 mg) (Sigma Aldrich) were added as internal standards for quantification of neutral sterols and bile acids, respectively. Samples were refluxed under nitrogen for 1 h, cooled and 1 mL of water and 5 mL of petroleum ether were added. Samples were mixed and centrifuged (2 min, 1000 × g). The petroleum ether and aqueous layers containing neutral sterols and bile acids, respectively, were further processed. The petroleum ether layer was collected, dried with sodium sulphate, transferred to a new tube and evaporated to dryness under nitrogen. Neutral sterols were derivatized by adding 0.5 mL of 1-(trimethylsilyl)imidazole (TMSI) + pyridine mixture (1:4, v/v) (92718, Sigma Aldrich) and incubating at 60°C for 1 h. Reagents were evaporated and derivatized neutral sterols were solubilized in 0.25 mL of hexane, centrifuged (2 min, 1000 × g) and the supernatant subjected to gas chromatography. For the extraction of bile acids, 0.5 mL of 10 N NaOH was added to the aqueous layer, the samples were heated at 120°C for 3 h, cooled and the sample acidified by adding 1 mL of concentrated HCL. Then, 7 mL of chloroform:methanol (2:1, v/v) was added, samples were mixed, centrifuged (2 min, 1000 × g) and the chloroform layer was collected. The chloroform was dried with sodium sulphate, transferred to a new tube and evaporated. Bile acids were methylated by adding 1 mL of dried methanol, 1 mL of dimethoxypropane, 20 μl of concentrated HCL and incubating at room temperature for 2 h in the dark. Reagents were evaporated and methylated bile acids were derivatized using TMSI + pyridine and subjected to gas chromatography as described for the neutral sterols.

Gas chromatography was performed using a 30-m x 0.25-mm capillary column (DB-1701, Agilent). The injector and the detector temperatures were 300°C. Neutral sterols were analyzed on an isothermal run at 270°C. For bile acids, the initial oven temperature was 240°C, followed by 2 ramps at 42 min (to 275°C) and 49 min (to 280°C). Neutral sterol and bile acid peaks were identified by comparing with known standards (Sigma Aldrich and Steraloids, Newport, RI, USA). Concentrations were determined by comparison with peaks of the corresponding internal standard.

### QPCR

Total liver RNA was isolated from the left lateral lobe, purified and DNase I treated using the RNeasy Mini kits (Qiagen, Mississauga, ON, Canada). RNA was quantified using a NanoDrop Spectrophotometer (ThermoScientific, Wilmington, DE, USA) and the integrity of each sample was verified by agarose gel electrophoresis. Two μg of total RNA was reverse transcribed using random primers using the High-Capacity cDNA Reverse Transcription Kit (Life Technologies, Carlsbad, CA, USA). QPCR was performed on a ViiA7 Quantitative PCR System (Applied Biosystems by Life Technologies, Austin, TX, USA) using TaqMan reagents and TaqMan gene expression assays for *Cyp7a1* (Rn00564065_m1), *Ldlr* (Rn00598442_m1), *Hmgcr* (Rn00565598_m1), *Hmgcs1* (Rn01493959_m1), *Fasn* (Rn00569117_m1), *Cpt2* (Rn00563995_m1), *Acat2* (Rn01526241_g1), and *18s* (Rn03928990_g1) (Applied Biosystems by Life Technologies). For each experiment, no template and no reverse transcriptase negative controls were included. The amounts of each gene-of-interest were determined using the standard curve method and normalized to 18s expression. Normalized values were calibrated to the 5Ca group (mean set as 1.00).

### Calculations

Apparent digestibility of total lipids and individual fatty acids was calculated from amounts consumed from the diet and amounts excreted in feces using the formula:

$$Digestibility\;\left(\%\right)=\left(Total\;consumed–Total\;excreted\right)\;{\left(Total\;consumed\right)}^{‑1}\times 100$$

Apparent cholesterol retained (CR) was calculated from amount of cholesterol consumed from the diet and amounts of neutral sterols (animal) and bile acids excreted in feces using the formula:

CR=Cholesterolconsumed–Neutralsterols(animal)excreted–Bileacidsexcreted

### Statistical analyses

Results are reported as means ± SD. Differences in means were determined by one-way ANOVA. When overall results were significant the Holm-Sidak post-hoc test was used to determine which means differed. Mixed-design ANOVA was used for analysis of parameters measured at multiple time points to determine the effects and interaction of diet and time. For time points with a significant diet effect differences among groups were determined by one-way ANOVA followed by the Holm-Sidak test. Homogeneity of variances was assessed using Levene’s test. Data that showed unequal variances were transformed prior to analysis. When equality of variances could not be achieved the non-parametric Kruskal–Wallis ANOVA and multiple comparisons of mean ranks were used to determine differences among groups. Statistical significance was set at *p* < 0.05. Data were analyzed using Statistica 7.1 (StatSoft, Tulsa, OK, USA) and SigmaPlot 12.5 (Systat Software, Inc., San Jose, CA, USA).

## Results

### Food intake and body composition

Rats were fed high-fat, high-energy diets containing different amounts of Ca (S1 Table). By analysis the diets contained 16% (0.75Ca), 42% (2Ca), 102% (5Ca, normal Ca), 202% (10Ca) and 402% (20Ca) of the normal Ca requirement of 5 g/kg diet for growing rats (55). Concentrations of total SFA, MUFA, PUFA, TFA and cholesterol did not differ among diets (S2 Table).

**Fig 1:**
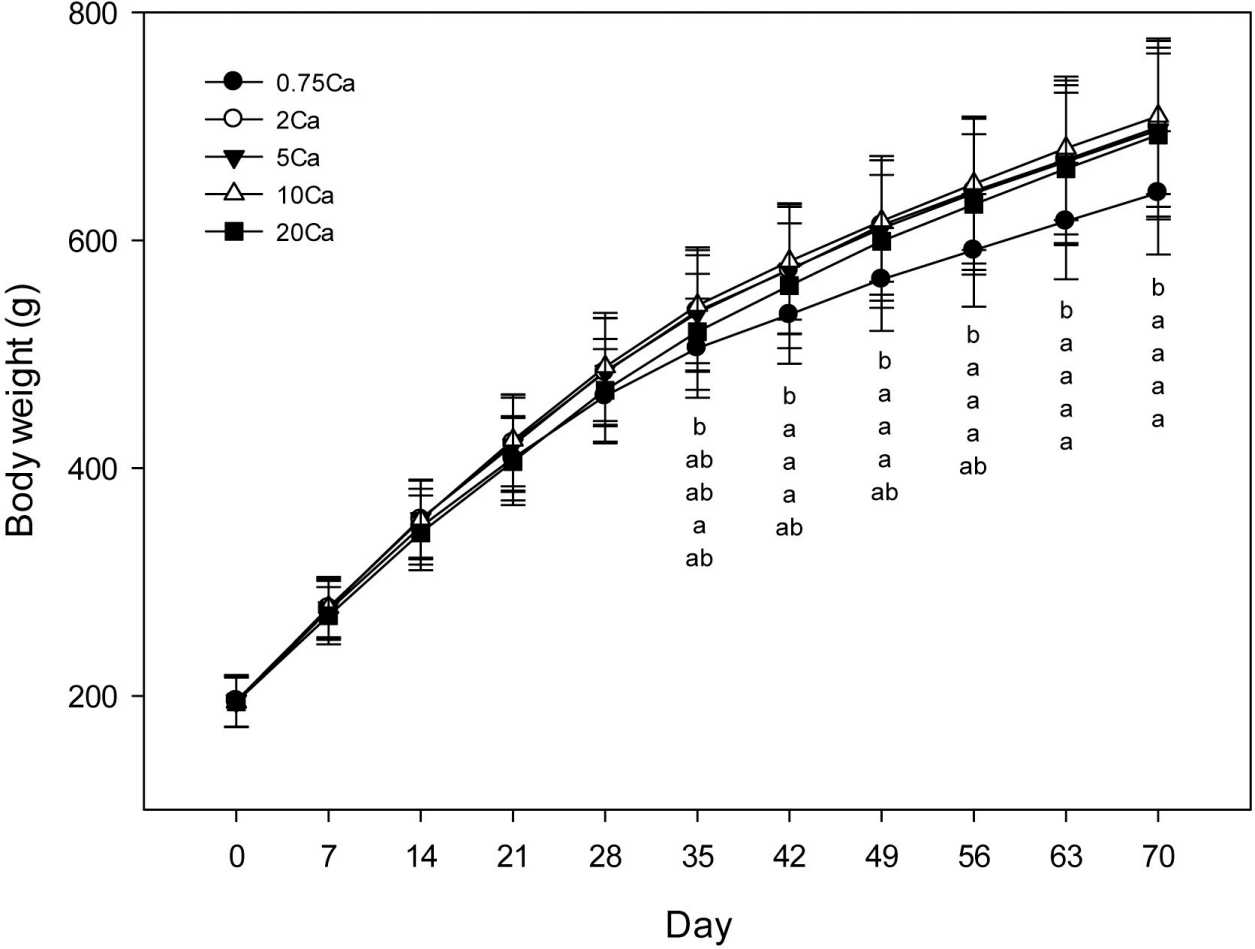
Body weights of rats. Results are presented as means ± SD, n = 28–30. Results were analyzed by mixed-design ANOVA to determine effects and interaction of diet and time. For time points with a significant (*p* < 0.05) diet effect, differences among diet groups were determined using one-way ANOVA followed by the Holm-Sidak post-hoc test. Diet groups without a common letter differ, *p* < 0.05. Letters correspond to diet groups 0.75Ca, 2Ca, 5Ca, 10Ca and 20Ca sequentially from top to bottom.

Body weights did not differ among the 2Ca, 5Ca, 10Ca or 20Ca groups at any time point during the study (Fig 1). At day 35, rats fed the 0.75Ca diet were lighter compared to rats fed the 10Ca diet and by the end of the study (Day 70) these rats were lighter compared to all other groups. Baseline measurements (Day 0) for percentage lean mass, total lean mass, percentage fat mass and total fat mass were similar among groups (Table 1). These measures did not differ among the 2Ca, 5Ca, 10Ca or 20Ca groups at Day 35 or Day 70. At Day70 rats fed the 0.75Ca diet had higher percentage lean mass and lower total fat mass compared to all other groups. The weights of inguinal, mesenteric and epididymal fat depots were lower for rats fed the 0.75Ca diet compared to rats fed the 5Ca, 10Ca or 20Ca diets. Weights of fat depots were similar among the 2Ca, 5Ca, 10Ca or 20Ca groups. Collectively, these results demonstrate that increases in dietary Ca from normal requirements did not affect body weight or body composition of the rats, but very low amounts of dietary Ca reduced body weight and fat mass.

**Table 1:** Body composition of rats.

Parameter	Diet groups				
	0.75Ca (n = 29)	2Ca (n = 28)	5Ca (n = 30)	10Ca (n = 30)	20Ca (n = 30)
Lean (%) ^1^					
Day 0	81.3 ± 2.1	81.3 ± 2.7	81.2 ± 2.3	81.1 ± 2.2	81.6 ± 2.5
Day 35	79.3 ± 3.8^a^	76.5 ± 4.5^ab^	75.3 ± 4.0^b^	74.1 ± 3.8^b^	76.1 ± 3.9^b^
Day 70	76.3 ± 4.9^a^	72.3 ± 4.6^b^	70.9 ± 5.0^b^	69.7 ± 3.9^b^	70.1 ± 4.5^b^
Lean (g) ^1^					
Day 0	164 ± 16	163 ± 15	163 ± 15	163 ± 15	164 ± 16
Day 35	400 ± 28	410 ± 29	403 ± 24	401 ± 28	394 ± 29
Day 70	488 ± 38	504 ± 43	491 ± 35	493 ± 42	484 ± 39
Fat (%) ^1^					
Day 0	11.9 ± 1.8	12.3 ± 2.0	12.3 ± 1.7	12.3 ± 1.8	11.8 ± 1.7
Day 35	17.3 ± 3.7^b^	19.4 ± 4.3^ab^	20.2 ± 4.1^a^	21.3 ± 3.7^a^	19.1 ± 3.6^ab^
Day 70	19.8 ± 4.6^b^	22.7 ± 4.5^ab^	23.9 ± 4.9^a^	25.1 ± 4.0^a^	24.3 ± 4.5^a^
Fat (g) ^1^					
Day 0	24.3 ± 6.1	25.1 ± 6.7	25.1 ± 6.0	25.0 ± 6.2	24.0 ± 6.0
Day 35	88.3 ± 23.9^b^	106 ± 32^ab^	110 ± 32^a^	117 ± 28^a^	101 ± 26^ab^
Day 70	128 ± 37^b^	161 ± 42^a^	169 ± 53^a^	179 ± 39^a^	170 ± 43^a^
Ing fat (g) ^2^	15.4 ± 5.7^b^	21.2 ± 6.8^a^	22.8 ± 10.8^a^	24.9 ± 7.5^a^	23.8 ± 8.2^a^
Retro + Peri fat (g) ^2^	22.4 ± 7.6^b^	27.6 ± 8.5^ab^	29.1 ± 8.3^a^	31.8 ± 7.8^a^	28.9 ± 7.9^a^
Mes fat (g) ^2^	7.0 ± 2.2^b^	9.8 ± 3.1^a^	10.1 ± 3.2^a^	10.5 ± 2.6^a^	10.8 ± 2.7^a^
Epi fat (g) ^2^	14.5 ± 3.8^b^	18.9 ± 4.9^a^	19.7 ± 5.1^a^	20.7 ± 4.5^a^	19.0 ± 4.8^a^

Values are means ± SD.

^1^ Analyzed by mixed-design ANOVA to determine effects and interaction of diet and time. For time points with a significant (*p* < 0.05) diet effect, differences among diet groups were determined using one-way ANOVA followed by the Holm-Sidak post-hoc test.

^2^ Analyzed by one-way ANOVA and Holm-Sidak test.

Values in a row without a common superscript letter differ, *p* < 0.05. Epi: epididymal; Ing: inguinal; Mes: mesenteric; Peri: perirenal; Retro: retroperitoneal.

**Fig 2:**
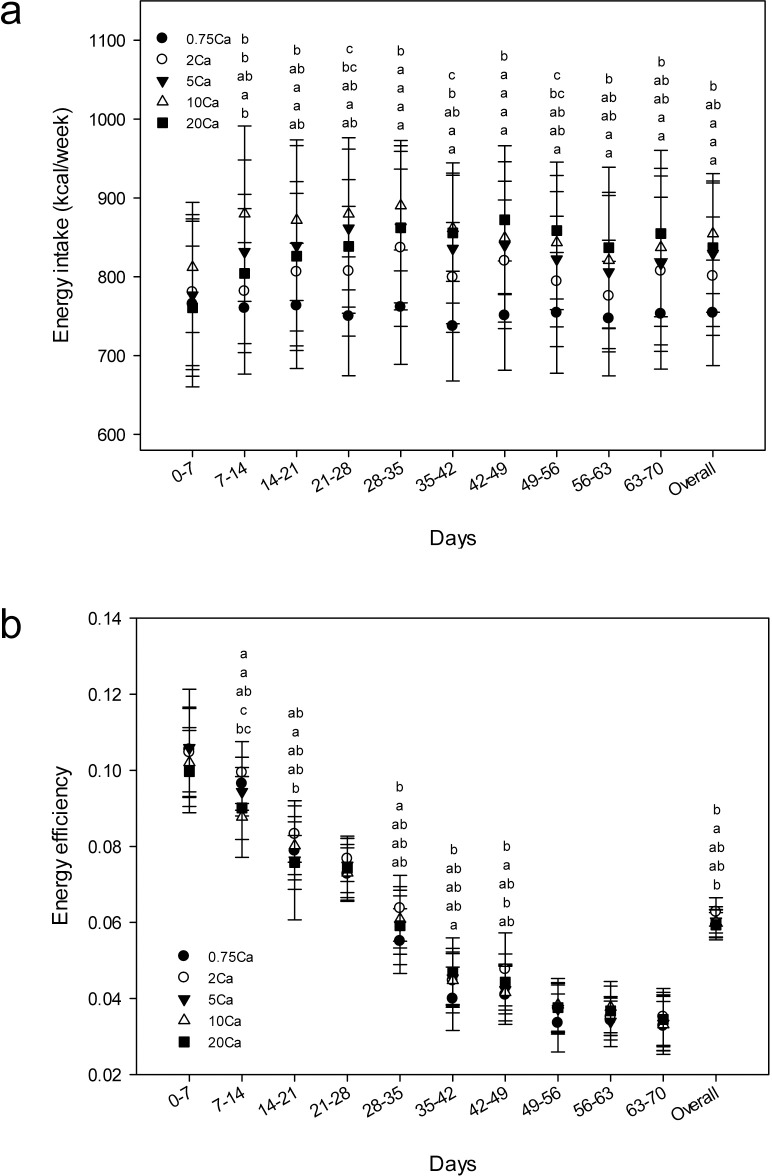
**Energy intake (a) and energy efficiency (b) of rats.** Results are presented as means ± SD, n = 28–30. Results were analyzed by mixed-design ANOVA to determine effects and interaction of diet and time. For time points with a significant (*p* < 0.05) diet effect, differences among diet groups were determined using one-way ANOVA followed by the Holm-Sidak post-hoc test. Diet groups without a common letter differ, *p* < 0.05. Letters correspond to diet groups 0.75Ca, 2Ca, 5Ca, 10Ca and 20Ca sequentially from top to bottom. Overall energy intake was calculated by dividing the total energy intake for the entire study by the number of weeks of the study (10 weeks). Energy efficiency = body weight gain (g/week)/energy intake (kcal/week). Overall energy efficiency was calculated by dividing the body weight gain for the entire study by the total energy intake.

Overall food consumption was lower for rats fed the low Ca diets (0.75Ca and 2Ca) compared to rats fed the high Ca diets (10Ca and 20Ca) (S1 Fig). Overall energy intake was lower for rats fed the 0.75Ca diet compared to rats fed the 5Ca or high Ca diets (Fig 2A). Overall energy efficiency was lower for rats fed the 0.75Ca or 20Ca diets compared to rats fed the 2Ca diet (Fig 2B).

### Mineral concentrations in serum and urine

Changes in mineral concentrations in serum and urine (normalized to creatinine) were observed (S3 Table). Serum Ca was higher in rats fed the 20Ca diet compared to rats fed the low Ca diets. In comparison to rats fed the 5Ca diet, serum magnesium (Mg) was lower in rats fed the 20Ca diet and higher in rats fed the 0.75Ca diet. Serum phosphorus (P) was higher in rats fed the 0.75Ca or 20Ca diets compared to rats fed the 2Ca, 5Ca or 10Ca diets. Serum potassium (K) was lower in rats fed the 20Ca diet compared to rats fed the low Ca diets. Urine Ca was higher in the 20Ca group compared to all other groups. Urine P decreased in a dose-dependent manner in rats fed diets with higher Ca.

### Serum lipid profile

Fasting serum TC and LDL-C was lower in rats fed the high Ca diets compared to rats fed the 5Ca diet (Table 2). Serum triglycerides were lower in the 0.75Ca group compared to the 5Ca group. TC:HDL-C and LDL-C:HDL-C ratios were lower in rats fed the high Ca diets compared to rats fed the 5Ca diet.

**Table 2:** Fecal excretion and blood lipid, insulin, glucose and PTH concentrations.

Parameter	Diet groups				
	0.75Ca (n = 29)	2Ca (n = 28)	5Ca (n = 30)	10Ca (n = 30)	20Ca (n = 30)
Serum TC (mmol/L)	1.92 ± 0.45^ab^	1.99 ± 0.35^a^	2.07 ± 0.35^a^	1.70 ± 0.36^bc^	1.61 ± 0.29^c^
Serum LDL-C (mmol/L)	0.26 ± 0.09^a^	0.24 ± 0.05^a^	0.24 ± 0.05^a^	0.18 ± 0.05^b^	0.16 ± 0.04^b^
Serum HDL-C (mmol/L)	0.54 ± 0.09	0.55 ± 0.07	0.60 ± 0.07	0.54 ± 0.09	0.57 ± 0.07
Serum TG (mmol/L)	0.86 ± 0.43^b^	1.14 ± 0.38^ab^	1.27 ± 0.50^a^	1.14 ± 0.46^ab^	1.06 ± 0.41^ab^
Serum TC:HDL-C ratio	3.50 ± 0.43^a^	3.58 ± 0.29^a^	3.47 ± 0.33^a^	3.12 ± 0.33^b^	2.83 ± 0.32^c^
Serum LDL-C:HDL-C ratio	0.47 ± 0.11^a^	0.44 ± 0.07^ab^	0.40 ± 0.07^b^	0.33 ± 0.06^c^	0.28 ± 0.07^d^
Plasma insulin (ng/mL)	0.67 ± 0.38^b^	0.96 ± 0.34^a^	1.28 ± 0.74^a^	1.27 ± 0.76^a^	1.39 ± 0.74^a^
Plasma glucose (mg/dL)	160 ± 24	165 ± 21	163 ± 21	162 ± 16	155 ± 17
Plasma PTH (ng/L) ^1^	137 ± 59^ab^	159 ± 74^a^	127 ± 45^ab^	109 ± 74^b^	63 ± 41^c^
Wk 3 fecal excretion (g) ^2^	6.8 ± 0.8^d^	7.0 ± 1.3^d^	10.1 ± 1.6^c^	14.1 ± 2.4^b^	18.3 ± 2.9^a^
Wk 8 Fecal excretion (g) ^2^	7.1 ± 0.8^d^	7.4 ± 0.9^d^	11.3 ± 1.5^c^	15.2 ± 2.2^b^	20.6 ± 2.8^a^

Values are means ± SD. Values in a row without a common superscript letter differ, *p* < 0.05.

^1^ n = 25–30.

^2^ Feces were collect for 7 consecutive days, freeze-dried and weighed.

HDL-C: HDL cholesterol; LDL-C: LDL cholesterol; PTH: parathyroid hormone; TC: total cholesterol; TG: triglycerides; Wk: week.

### Plasma insulin, glucose and PTH

Plasma insulin concentration was lower in the 0.75Ca group compared to all other groups (Table 2). Plasma glucose concentration did not differ among groups. Plasma PTH concentration was lower in the 20Ca group compared to all other groups. Dry weights of feces excreted during week 3 or week 8 of the study increased in a dose-dependent manner in rats fed diets with higher Ca (Table 2).

### Liver weight and lipid concentrations

Liver weights (as a percentage of body weight) were lower in rats fed the high Ca diets and higher in rats fed the 2Ca diet compared to rats fed the 5Ca diet (Table 3). Liver total lipid concentration was higher for the 2Ca group compared to the high Ca groups. Liver TC concentration was lower in the 20Ca group compared to the 5Ca or low Ca groups. Liver free cholesterol or triglyceride concentrations did not differ among groups.

**Table 3:** Liver weight and lipid concentrations.

Parameter	Diet groups				
	0.75Ca (n = 29)	2Ca (n = 28)	5Ca (n = 30)	10Ca (n = 30)	20Ca (n = 30)
Liver weight (% BW)	2.91 ± 0.29^ab^	3.04 ± 0.32^a^	2.75 ± 0.29^b^	2.45 ± 0.29^c^	2.30 ± 0.19^c^
Total lipids (mg/g)	114 ± 33^ab^	137 ± 41^a^	116 ± 35^ab^	106 ± 34^b^	101 ± 23^b^
TC (mg/g)	5.02 ± 0.89^a^	4.86 ± 1.11^a^	4.45 ± 0.99^ab^	3.91 ± 0.99^bc^	3.74 ± 0.66^c^
FC (mg/g)	2.16 ± 0.45	2.07 ± 0.37	2.01 ± 0.49	2.04 ± 0.41	1.91 ± 0.22
TG (mg/g)	48.3 ± 20.4	55.1 ± 25.7	51.1 ± 26.8	44.2 ± 27.4	39.1 ± 16.3

Values are means ± SD. Values in a row without a common superscript letter differ, *p* < 0.05. BW: body weight; FC: free cholesterol; TC: total cholesterol; TG: triglycerides.

### Fatty acid digestibility

Fecal excretion and apparent digestibility of total lipids and fatty acids was determined during week 3 of the study. Amounts of total lipids and total SFA, MUFA, PUFA and TFA excreted was many fold higher in rats fed the 5Ca diet compared to rats fed the low Ca diets (0.75Ca and 2Ca) (S4 Table). Fecal excretion was even higher in rats fed the high Ca diets (10Ca and 20Ca) compared to rats fed the 5Ca diet. Digestibility of total lipids was 98% in rats fed the low Ca diets, 93% in rats fed the 5Ca diet and 89% in rats fed the high Ca diets (Table 4). Dietary Ca affected the digestibility of all 4 fatty acid classes. The largest effect was observed for TFA with a reduction from 98% in rats fed the low Ca diets to 42–46% in rats fed the high Ca diets. Digestibility of the primary natural (ruminant) TFA 18:1 11t was 92% in rats fed the low Ca diets and –144 to –120% in rats fed the high Ca diets. Digestibility of the most common industrial TFA 18:1 9t and 18:1 10t decreased from 99% in rats fed the low Ca diets to 85–86% in rats fed the high Ca diets. The digestibility of total conjugated linoleic acid (CLA) decreased from 98% to 44–49% in rats fed the low or high Ca diets, respectively. Comparable digestibility was observed for a naturally occurring isomer of CLA 18:2 9c, 11t.

**Table 4:** Apparent total lipid and fatty acid digestibility during week 3 of the study.

Lipid	Diet groups				
	0.75Ca (n = 29)	2Ca (n = 28)	5Ca (n = 30)	10Ca (n = 30)	20Ca (n = 30)
	Digestibility (%)				
12:0	100 ± 0^a^	100 ± 0^a^	98 ± 1^b^	97 ± 1^c^	97 ± 1^c^
14:0	100 ± 0^a^	100 ± 0^a^	96 ± 1^b^	93 ± 2^c^	93 ± 2^bc^
I15:0	93 ± 1^b^	94 ± 2^a^	90 ± 2^c^	87 ± 4^d^	85 ± 2^e^
15:0	96 ± 1^a^	96 ± 1^a^	86 ± 3^b^	82 ± 4^c^	81 ± 3^c^
I16:0	95 ± 1^a^	95 ± 2^a^	88 ± 3^b^	82 ± 5^bc^	81 ± 4^c^
16:0	100 ± 0^a^	100 ± 0^a^	86 ± 4^b^	80 ± 6^bc^	80 ± 5^c^
I17:0	99 ± 0^a^	98 ± 1^a^	89 ± 3^b^	82 ± 5^c^	81 ± 4^c^
17:0	99 ± 0^a^	99 ± 0^a^	82 ± 5^b^	75 ± 6^b^	75 ± 6^b^
18:0	99 ± 0^a^	99 ± 0^a^	75 ± 7^b^	69 ± 8^b^	68 ± 7^b^
I18:0	85 ± 4^a^	85 ± 8^a^	37 ± 18^b^	12 ± 26^bc^	5 ± 16^c^
20:0	98 ± 0^a^	98 ± 1^a^	71 ± 7^b^	63 ± 9^b^	62 ± 8^b^
22:0	92 ± 2^a^	92 ± 2^a^	62 ± 8^b^	54 ± 10^bc^	51 ± 7^c^
23:0	86 ± 3^a^	86 ± 4^a^	60 ± 8^b^	52 ± 9^b^	51 ± 7^b^
24:0	86 ± 3^a^	85 ± 4^a^	43 ± 11^b^	32 ± 13^bc^	26 ± 9^c^
Total SFA	99 ± 0^a^	99 ± 0^a^	85 ± 4^b^	79 ± 5^b^	79 ± 5^b^
16:1 9c	100 ± 0^a^	100 ± 0^a^	99 ± 0^b^	97 ± 1^c^	97 ± 1^c^
16:1 11c	97 ± 1^a^	97 ± 1^a^	93 ± 2^b^	91 ± 3^bc^	90 ± 3^c^
16:1 13c	89 ± 3^b^	91 ± 2^a^	85 ± 3^c^	81 ± 4^d^	79 ± 4^d^
17:1 9c	73 ± 7^a^	76 ± 6^a^	60 ± 7^b^	56 ± 10^b^	58 ± 5^b^
18:1 9c	100 ± 0^a^	100 ± 0^a^	98 ± 1^b^	94 ± 3^c^	94 ± 3^c^
18:1 11c	99 ± 0^a^	99 ± 0^a^	95 ± 1^b^	89 ± 4^c^	89 ± 3^c^
18:1 12c	100 ± 0^a^	100 ± 0^a^	93 ± 4^b^	92 ± 3^b^	91 ± 3^b^
18:1 13c	98 ± 0^a^	98 ± 1^a^	88 ± 5^b^	84 ± 6^b^	82 ± 5^b^
18:1 14c	98 ± 0^a^	98 ± 1^a^	73 ± 7^b^	61 ± 11^b^	62 ± 7^b^
18:1 15c	100 ± 0^a^	100 ± 0^a^	89 ± 4^b^	84 ± 5^b^	85 ± 4^b^
Total 18:1 cis	100 ± 0^a^	100 ± 0^a^	98 ± 1^b^	94 ± 3^c^	94 ± 3^c^
20:1 11c	99 ± 0^a^	99 ± 0^a^	94 ± 2^b^	86 ± 5^c^	86 ± 5^c^
22:1 13c	93 ± 2^a^	94 ± 1^a^	68 ± 8^b^	43 ± 14^c^	30 ± 12^c^
24:1 15c	84 ± 3^a^	86 ± 4^a^	68 ± 7^b^	63 ± 8^bc^	50 ± 9^c^
Total MUFA	100 ± 0^a^	100 ± 0^a^	98 ± 1^b^	94 ± 3^c^	94 ± 3^c^
18:2 9c, 11t	99 ± 1^a^	98 ± 1^a^	93 ± 5^b^	47 ± 54^c^	43 ± 70^c^
18:2 9t, 11t	97 ± 2^a^	98 ± 2^a^	91 ± 4^b^	48 ± 44^c^	42 ± 80^c^
18:2 10t, 12c	96 ± 2^ab^	97 ± 1^a^	93 ± 2^b^	69 ± 29^c^	64 ± 59^c^
Total CLA	98 ± 1^a^	98 ± 1^a^	92 ± 4^b^	49 ± 47^c^	44 ± 72^c^
18:2 n-6	100 ± 0^a^	100 ± 0^a^	99 ± 0^b^	99 ± 1^c^	99 ± 1^c^
20:2 n-6	96 ± 1^a^	97 ± 1^a^	91 ± 2^b^	86 ± 5^c^	84 ± 5^c^
20:3 n-6	91 ± 4^b^	95 ± 3^a^	92 ± 4^b^	82 ± 9^c^	79 ± 8^c^
20:4 n-6	86 ± 5^a^	86 ± 7^a^	79 ± 7^a^	53 ± 18^b^	47 ± 16^b^
22:4 n-6	87 ± 3^b^	90 ± 2^a^	85 ± 4^b^	76 ± 7^c^	65 ± 9^d^
Total n-6 PUFA	100 ± 0^a^	100 ± 0^a^	99 ± 0^b^	99 ± 1^c^	98 ± 1^c^
18:3 n-3	99 ± 0^a^	99 ± 0^a^	97 ± 1^b^	93 ± 2^c^	93 ± 2^c^
22:5 n-3	98 ± 1^a^	99 ± 1^a^	98 ± 2^a^	95 ± 2^b^	94 ± 4^b^
Total n-3 PUFA	99 ± 0^a^	99 ± 0^a^	95 ± 1^b^	91 ± 3^c^	90 ± 2^c^
Total PUFA	100 ± 0^a^	100 ± 0^a^	99 ± 0^b^	98 ± 1^c^	98 ± 1^c^
18:1 (6t-8t)	100 ± 0^a^	99 ± 0^a^	82 ± 11^b^	87 ± 5^b^	86 ± 4^b^
18:1 9t	99 ± 0^a^	99 ± 0^a^	90 ± 4^b^	86 ± 5^b^	85 ± 4^b^
18:1 10t	99 ± 0^a^	99 ± 1^a^	88 ± 5^b^	86 ± 6^b^	85 ± 5^b^
18:1 11t	92 ± 4^a^	92 ± 4^a^	26 ± 45^b^	-144 ± 112^c^	-120 ± 96^c^
18:1 12t	99 ± 0^a^	99 ± 0^a^	91 ± 3^b^	86 ± 5^bc^	85 ± 4^c^
18:1 (13t+14t)	100 ± 0^a^	100 ± 0^a^	87 ± 4^b^	81 ± 6^b^	81 ± 5^b^
18:1 16t	100 ± 0^a^	100 ± 0^a^	87 ± 4^b^	81 ± 5^bc^	80 ± 5^c^
Total 18:1 TFA	97 ± 1^a^	97 ± 1^a^	71 ± 15^b^	21 ± 34^c^	27 ± 29^c^
18:2 9c, 12t	100 ± 0^a^	100 ± 0^a^	98 ± 1^b^	95 ± 2^c^	95 ± 2^c^
18:2 9t, 12c	98 ± 1^a^	97 ± 1^a^	89 ± 3^b^	85 ± 5^bc^	82 ± 5^c^
Total 18:2 TFA	99 ± 0^a^	99 ± 0^a^	95 ± 1^b^	92 ± 3^bc^	91 ± 2^c^
18:3 9t, 12c, 15c	100 ± 0^a^	100 ± 0^a^	95 ± 2^b^	87 ± 5^c^	87 ± 5^c^
Total TFA	98 ± 1^a^	98 ± 1^a^	78 ± 11^b^	42 ± 24^c^	46 ± 21^c^
Total lipids	98 ± 0^a^	98 ± 1^a^	93 ± 2^b^	89 ± 3^c^	89 ± 3^c^

Values are means ± SD. Values in a row without a common superscript letter differ, *p* < 0.05. CLA: conjugated linoleic acid; MUFA: monounsaturated fatty acids; PUFA: polyunsaturated fatty acids; SFA: saturated fatty acids; TFA: trans fatty acids.

Digestibility of total SFA decreased from 99% in rats fed the low Ca diets to 79–85% in rats fed the 5Ca or high Ca diets. Compared to rats fed the 5Ca diet, digestibility of lauric (12:0) and myristic (14:0) acid was lower in rats fed the 10Ca diet and digestibility of lauric and palmitic (16:0) acid was lower in rats fed the 20Ca diet. In general, Ca had a greater effect on the digestibility of longer chain SFA. For SFA with a carbon chain length ≥ 20 the digestibility was 26–63% in rats fed the high Ca diets compared to 85–98% in rats fed the low Ca diets.

Compared to TFA or SFA, the digestibility of total MUFA and PUFA were less affected by the Ca content in the diets. Total MUFA digestibility decreased from 100% in rats fed the low Ca diets to 94% in rats fed the high Ca diets. The corresponding digestibility for total PUFA was 100% and 98%.

Dietary Ca had only a small effect on the digestibility of total n-6 PUFA or linoleic acid (18:2 n-6) as evidenced by a digestibility of ≥ 98% in rats fed the high Ca diets. Longer chain n-6 PUFA were more affected by dietary Ca with digestibility of 47–86% in rats fed the high Ca diets. Total n-3 PUFA digestibility was reduced from 99% in rats fed the low Ca diets to 90–91% in rats fed the high Ca diets. Comparable digestibility was observed for α-linolenic acid (18:3 n-3).

### Neutral sterol and bile acid excretion

Fecal excretion of neutral sterols and bile acids was determined during week 8 of the study. Fecal excretion of cholesterol was higher in rats fed the 20Ca diet compared to all other groups (Table 5). Compared to the 5Ca group, excretion of total animal sterols (cholesterol, coprostanol and cholestenol) was higher in rats fed the 20Ca diet and lower in rats fed the low Ca diets. Excretion of total plant sterols (β-sitosterol and stigmasterol) was higher in rats fed the 20Ca diet compared to rats fed the 5Ca diet. Excretion of total bile acids was higher in the 20Ca group compared to all other groups. Apparent cholesterol retained (calculated as the difference between cholesterol intake and the sum of total animal neutral sterols and total bile acids excreted) was lower for rats fed the 20Ca diet and higher for rats fed the low Ca diets compared to rats fed the 5Ca diet. Notably, cholesterol retained did not differ between rats fed the 5Ca or 10Ca diets. The negative values indicate, for all diet groups, greater loss of cholesterol from excretion of cholesterol and cholesterol metabolites compared to amounts of cholesterol consumed from the diet.

**Table 5:** Fecal excretion of neutral sterols and bile acids during week 8 of the study.

Parameter			Diet groups		
	0.75Ca (n = 9)	2Ca (n = 9)	5Ca (n = 9)	10Ca (n = 9)	20Ca (n = 9)
Fecal NS excretion (mg/wk)					
cholesterol	47.9 ± 9.1^c^	56.2 ± 10.0^bc^	51.3 ± 8.2^bc^	65.0 ± 8.2^b^	106 ± 38^a^
coprostanol	7.19 ± 7.34^b^	9.86 ± 6.63^b^	38.4 ± 10.9^a^	31.3 ± 11.8^a^	15.5 ± 11.0^b^
cholestanol	0.82 ± 0.24	0.97 ± 0.20	0.93 ± 0.17	0.99 ± 0.26	1.21 ± 0.59
Total animal NS excretion	55.9 ± 9.5^c^	67.0 ± 9.7^c^	90.7 ± 14.8^b^	97.3 ± 12.3^ab^	123 ± 34^a^
β-sitosterol	83.1 ± 22.7^b^	93.9 ± 17.9^ab^	53.6 ± 14.0^b^	72.8 ± 14.9^b^	133 ± 65^a^
stigmasterol	9.15 ± 2.12^b^	10.2 ± 2.0^ab^	6.54 ± 1.41^b^	8.27 ± 1.39^b^	14.3 ± 6.9^a^
Total plant NS excretion	92.3 ± 24.8^b^	104 ± 20^ab^	60.2 ± 15.4^b^	81.1 ± 16.3^b^	147 ± 72^a^
Fecal BA excretion (mg/wk)					
α-muricholic acid	2.99 ± 1.24^b^	2.44 ± 1.00^b^	3.11 ± 1.43^b^	2.98 ± 1.06^b^	6.53 ± 2.65^1^^, a^
β-muricholic acid	16.7 ± 6.2	17.5 ± 10.6	14.6 ± 5.3	10.8 ± 3.8	23.0 ± 21.5^1^
γ-muricholic acid	1.32 ± 0.55^b^	0.84 ± 0.33^b^	1.09 ± 0.47^b^	1.27 ± 0.35^b^	2.47 ± 1.21^1^^, a^
cholic acid	3.76 ± 2.38^a^	1.61 ± 1.25^b^	0.65 ± 0.21^b^	1.22 ± 0.69^b^	3.53 ± 1.96^1^^, a^
deoxycholic acid	4.93 ± 1.73^b^	4.88 ± 1.98^b^	13.3 ± 2.7^a^	15.1 ± 5.2^a^	17.8 ± 6.3^1, a^
chenodeoxycholic acid	0.74 ± 0.52^b^	0.67 ± 0.55^b^	0.71 ± 0.25^b^	1.07 ± 0.66^ab^	1.87 ± 1.18^1^^, a^
lithocholic acid	1.47 ± 0.51^b^	1.22 ± 0.41^b^	4.17 ± 1.63^a^	4.47 ± 1.78^a^	5.35 ± 2.33^1^^, a^
Total BA excretion	31.9 ± 9.6^b^	29.2 ± 11.7^b^	37.5 ± 9.0^b^	36.9 ± 8.9^b^	60.6 ± 21.7^1^^, a^
CR (mg/wk)	–56.7 ± 17.4^a^	–61.6 ± 17.0^a^	–93.0 ± 15.7^b^	–97.8 ± 11.8^b^	–142 ± 55^1^^, c^

Values are means ± SD. Values in a row without a common superscript letter differ, *p* < 0.05.

^1^ n = 8.

CR = Cholesterol intake–Neutral sterols (animal) excreted–Bile acids excreted. BA: bile acid; CR: cholesterol retained; NS: neutral sterol; wk: week.

### mRNA expression of lipogenic enzymes in liver

Dietary Ca altered liver lipid concentrations. Thus, we examined mRNA expression of lipogenic genes in the liver of the rats (S5 Table). Expression of *Cyp7a1* that functions in bile acid synthesis was similar among diet groups. Expressions of *Ldlr* (LDL clearance from the circulation), *Hmgcr* and *Hmgcs1* (cholesterol synthesis), *Cpt2* (fatty acid oxidation) and *Acat2* (cholesterol esterification) were higher in rats fed diets with higher Ca. *Fasn* (fatty acid synthesis) expression was lower in rats fed the low Ca diets compared to rats fed the 5Ca or high Ca diets.

## Discussion

Randomized controlled trials (RCT) and meta-analyses of RCT of Ca supplementation (with or without vitamin D) have reported a modest increase in risk of cardiovascular events, in particular myocardial infraction, in participants taking a Ca supplement (500–1000 mg/day) compared to placebo [12–14]. However, the totality of available evidence to date does not seem to support a causal inference between higher dietary Ca or Ca supplementation and increased risk of cardiovascular events [5, 62]. A recent updated systematic review and meta-analysis concluded that Ca intakes below the upper limit (i.e., 2000–2500 mg/day) were not associated with increased risk of CVD events or mortality in healthy adults [63]. Furthermore, there is some evidence suggesting that higher Ca intakes may lower CVD risk by promoting lower body weight or fat mass and positively altering serum lipid profile [6–11].

In this study, rats fed different amounts of Ca showed expected changes in plasma PTH and serum and urine mineral concentrations. Rats fed the highest Ca diet (20Ca) had lower plasma PTH and higher urine Ca concentrations compared to other groups. Serum Ca concentrations of the 20Ca group were higher compared to rats fed the low Ca diets (0.75Ca and 2Ca). These results are consistent with the decrease in PTH secretion in response to higher serum Ca [3]. Lower circulating PTH attenuates increases in serum Ca by increasing urinary Ca excretion and decreasing resorption of Ca from bone [3].

Metabolism of Ca, P and Mg are interconnected. Serum P concentrations were higher in rats fed the highest (20Ca) and lowest (0.75Ca) Ca diets compared to rats fed normal Ca (5Ca). These differences may be explained by effects of dietary Ca on urinary P excretion, intestinal absorption and resorption from bone. Lower circulating PTH concentrations in response to increases in serum Ca decrease urinary P excretion [3]. Urine P concentrations were decreased in a dose-dependent manner in rats fed higher Ca. This reduction in urinary P may have contributed to the higher serum P in rats fed the 20Ca diet. The higher serum P in rats fed the 0.75Ca diet may be explained by higher intestinal absorption of P and possibly increased P resorption from bone. These effects may have outweighed the higher urinary P excretion in rats fed the 0.75Ca diet. The decline in serum Mg concentrations in rats fed higher Ca is most likely explained by increased competition for intestinal absorption between Ca and Mg given the lack of differences in urinary Mg excretion among groups [64].

Results from human and animal studies examining effects of Ca intakes on body weight and fat mass have been mixed [11, 15–36]. In an effort to reconcile results, it has been proposed that higher Ca intakes may only have a small effect on reducing body weight and fat mass and larger effects may only occur in specific circumstances such as conditions of energy restriction or when baseline Ca status is low [7]. It has also been suggested that dairy products may have a larger effect compared to Ca alone that may be explained by additional anti-obesity compounds in dairy [9]. In this study diets containing between ~40% (2Ca) and 400% (20Ca) of normal Ca, fed *ad libitum*, did not affect body weight or fat mass of the rats. Rats fed the 0.75Ca diet containing very low Ca (~16% of normal) had lower body weight and fat mass compared to other groups that may be explained by lower food consumption and energy intake. It should be mentioned, however, that this degree of Ca deficiency is rare in the general population.

It has been proposed that higher Ca intakes may promote weight loss by decreasing appetite or decreasing fat absorption and consequently digestible energy [7]. Overall food consumption was greater in rats fed the high (10Ca and 20Ca) compared to the low (0.75Ca and 2Ca) Ca diets. These differences may be explained by the modest differences in energy densities of the diets. This is supported by results demonstrating similar energy intakes among groups (excluding the 0.75Ca group that had lower body weight). Overall energy efficiency was lower for rats fed the highest Ca diet (20Ca) compared to rats fed the 2Ca low Ca diet. This may be explained in part by the lower dietary fat digestibility and presumably lower energy digestibility of the 20Ca diet. Collectively, the results suggest that the higher Ca diets did not reduce appetite of the rats. These results are in general agreement with a study in diet-induced obese mice that reported higher food consumption and increased body weight and fat depots with higher dietary Ca [33]. In that study, only mice fed a diet containing high Ca plus non-fat dry milk showed reduced body weight and adiposity compared to control mice fed a normal Ca diet suggesting other components in the dairy (not the Ca) had anti-obesity properties. Results from the current study also do not support the premise that lower fat digestibility of diets higher in Ca decrease body weight or fat mass in rats.

Some research has suggested that higher Ca intakes decrease lipogenesis and increase lipolysis in adipose tissue leading to reduced body fat [11, 23]. The proposed mechanism involves a decrease in circulating PTH and 1,25 vitamin D which in turn decreases intracellular Ca concentrations. In this study, rats fed the 20Ca diet had lower circulating PTH (1,25 vitamin D was not measured) compared to other groups, but these rats did not have lower total fat mass measured by magnetic resonance imaging nor weights of 4 distinct fat depots. Therefore, if the differences in dietary Ca affected lipogenesis or lipolysis in adipose tissue, the effects were small and physiologically irrelevant.

Rats fed high Ca (10Ca and 20Ca) had lower serum TC and LDL-C as well as TC:HDL-C and LDL-C:HDL-C ratios compared to rats fed normal Ca (5Ca). Abnormal glucose metabolism and insulin resistance is associated with elevated serum concentrations of triglyceride-rich lipoproteins and reduced HDL cholesterol [65]. An inverse relationship between Ca intake and plasma glucose and insulin concentrations has been reported in rats [29]. In this study plasma glucose and insulin concentrations did not differ among rats fed between ~40% (2Ca) and 400% (20Ca) of normal Ca suggesting little effect of dietary Ca at these doses on glucose metabolism and insulin sensitivity. Thus it is unlikely that changes in glucose metabolism or insulin sensitivity accounted for the observed differences in serum lipids of the rats. Rats fed very low Ca (0.75Ca) had lower serum insulin compared to the other groups that may be explained by impaired secretion of insulin from pancreatic β-cells [66].

Compared to rats fed normal Ca (5Ca), liver weight (normalized to body weight) was lower in rats fed the high Ca diets (10Ca and 20Ca) and TC concentrations in the liver were lower in rats fed the highest Ca diet (20Ca). Total lipid concentrations in the liver showed a decreasing trend in rats fed higher Ca, but concentrations did not differ significantly (*p* ≥ 0.05) among rats fed the 5Ca, 10Ca or 20Ca diets. Therefore, differences in lipid accumulation in the liver may not entirely account for the differences in liver weights among groups.

Dietary Ca induced changes in mRNA expression of lipogenic genes in the liver. Rats fed higher Ca had higher expression of *Cpt2* suggesting an increase in mitochondrial fatty acid oxidation. Rats fed the low Ca diets (0.75Ca and 2Ca) had lower expression of *Fasn* compare to rats fed the normal (5Ca) or high (10Ca and 20Ca) Ca diets suggesting decreased endogenous fatty acid synthesis in rats fed a Ca-deficient diet. In general, expression of the *Ldlr* that functions in hepatic cholesterol uptake from the circulation and genes involved in endogenous cholesterol synthesis (*Hmgcr* and *Hmgcs1*) and cholesterol esterification (*Acat2*) were higher in rats fed higher Ca. Higher expression of these genes is consistent with a response by the liver to a direct or indirect depletion of liver cholesterol. Compared to rats fed normal Ca (5Ca), fecal excretion of cholesterol, total neutral sterols (sum of cholesterol, coprostanol and cholestanol) and total bile acids was higher, while apparent cholesterol retained was lower in rats fed the highest Ca diet (20Ca). These results suggest a greater loss of cholesterol in rats fed the 20Ca diet which may have contributed to the lower serum TC and LDL-C. A decrease in liver cholesterol may have triggered an upregulation of the LDL receptor increasing LDL uptake from the circulation to compensate for the increased demand for cholesterol for the synthesis of bile acids. A study in mice has suggested that a direct intestinal cholesterol secretion pathway may be induced to compensate for decreased biliary cholesterol secretion [67]. The lower liver cholesterol in rats fed the 20Ca diet may have decreased biliary bile acid and cholesterol secretion and consequently increased cholesterol excretion from intestinal cells. Thus, we cannot exclude the possibility that upregulation of the direct intestinal secretion pathway for cholesterol contributed to the higher fecal cholesterol/neutral sterol excretion and changes in serum lipids in rats fed the 20Ca diet. A study has reported differences in mRNA expression of intestinal genes involved in cholesterol metabolism including *NPC1L1*, *MTP* and *ABCG5/8* in ovariectomized hamsters fed diets with different amounts of Ca [41]. It will be interesting to determine in future studies whether changes in intestinal expression of these genes contributed to the differences in fecal cholesterol excretion observed for rats fed different levels of dietary Ca in this study. Notably, although values for liver TC concentrations and apparent cholesterol retained were numerically lower in rats fed the 10Ca compared to the normal (5Ca) Ca diet differences did not reach statistical significance. This suggests there may be an alternative mechanism to explain the differences in serum lipids between these groups.

Ca can bind and precipitate fatty acids in the GI tract reducing their absorption and increasing excretion in feces. If Ca preferentially reduces the absorption of SFA or TFA compared to MUFA or PUFA, higher intakes of Ca could be expected to have a beneficial effect on serum lipid profile. The basal diet in this study contained a ruminant source of fat (anhydrous milk-fat) which allowed for the investigation of the effects of Ca on all 4 major classes of fatty acids including TFA. TFA originate from natural (ruminant) or industrial (formed during hydrogenation processes or inadvertently during oil refining) sources. It is well-established that industrial TFA have a negative effect on serum lipids and pose a health risk, but whether TFA as part of dairy products also pose a health risk is currently a topic of debate [52].

Fecal excretion of all 4 classes of fatty acids increased dramatically when rats were fed higher Ca. Rats fed diets with higher Ca also showed decreases in the apparent digestibility of the 4 classes of fatty acids. The largest effects on digestibility were seen for TFA followed by SFA. The differences in total TFA digestibility were substantial. Rats fed low, normal or high Ca had digestibilities of 98%, 78% and 42–46%, respectively. It is possible that the lower digestibility of TFA in rats fed the high Ca diets contributed to the lower serum TC and LDL-C in these rats given the positive relationship between intake of TFA and blood cholesterol [52].

Digestibility of all TFA were affected by Ca with the largest effect seen for vaccenic acid (18:1 11t), the primary natural TFA. The negative values for the digestibility of vaccenic acid in rats fed the high Ca diets indicate that more of the fatty acid was excreted in the feces than consumed from the diet. This is likely explained by the conversion of other fatty acids such as conjugated linoleic acid (CLA) to vaccenic acid by intestinal bacteria [68].

The higher Ca diets reduced the digestibility of SFA and to a lesser degree the digestibility of MUFA and PUFA. The greater effect of Ca on digestibility of SFA may be explained by the slower absorption of saturated compared to unsaturated fatty acids. Longer resident time in the gut may make SFA more susceptible to precipitation by Ca. Generally, larger effects were seen for longer chain SFA that may be explained by lower solubility of longer chain fatty acids when complexed with Ca. It is important to mention that although digestibility of total SFA did not differ significantly among rats fed the normal or high Ca diets, digestibility of the hypercholesterolemic SFA lauric, myristic and palmitic acid were lower in rats fed the high Ca diets.

The main limitation of this study is that the effects of dietary Ca on pathological endpoints of CVD were not examined. The effects on blood lipids suggest reduced risk for CVD in rats fed high Ca but we cannot say for certain that the high Ca diets were protective of CVD. More research on the effects of higher intakes of Ca on CVD endpoints is warranted. Also, the effects of Ca on fatty acid digestibility and excretion of neutral sterols and bile acids were determined in fecal samples collected over a 1-week period of a 10-week study. However, differences in fecal bulk among diet groups measured during weeks 3 and 8 were comparable suggesting that the effects of Ca was likely uniform throughout the study. Although rat models are commonly used in obesity and lipid research, differences in lipid metabolism exist between rats and humans [69]. Notably, rats transport most cholesterol in HDL particles (instead of LDL particles) and lack plasma cholesteryl ester transfer protein (CETP) activity. A main strength of the study is that fatty acid digestibility (including TFA), fecal excretion of neutral sterols and bile acids and lipid metabolism in the liver were investigated in the same study across multiple doses of Ca. This experimental approach provides a better indication of the specific doses of Ca and underlying mechanisms driving changes in blood lipids.

## Conclusions

Ca intakes for a large segment of the North American population fall short of dietary recommendations [70, 71], while upper percentiles of intakes for some subpopulations (e.g., older women) exceed the upper limit from use of high-dose Ca supplements [72]. Thus, it is important to understand the effects of Ca intakes on CVD risk. This study has shown that differences in dietary Ca alone (i.e., without changes in other components in dairy foods) has little effect on body weight or fat mass in rats unless Ca intakes are very low. Diets containing above normal amounts of Ca induced changes in blood lipids predictive of a lower risk for CVD. More than one mechanism may account for the changes in blood lipids. At high Ca doses (20Ca diet containing ~400% of normal Ca), decreased absorption of neutral sterols and increased excretion of bile acids as well as decreased digestibility of TFA and SFA may have contributed to the lower serum TC and LDL-C. At a lower Ca dose above normal (10Ca diet containing ~200% of normal Ca), decreased digestibility of TFA and SFA may have played a larger role given the absence of a significant effect on cholesterol balance. Ca intakes of 200% normal requirements (i.e., ~2-fold the recommended dietary allowance) occur in the general population. Consumption of 400% normal Ca requirements is rare, but high concentrations of Ca in the GI tract can occur with consumption of high-dose Ca supplements at one time. A major finding reported in this paper is the large decrease in digestibility of TFA with higher dietary Ca. Further research is needed to determine whether the Ca in dairy products attenuates the adverse effects of TFA in these products. This is important since natural TFA may have the same adverse effects on blood lipids as industrial TFA, but are more difficult to remove from the food supply.

## Supporting information

S1 Table Diet compositions. (PDF)

S2 Table Analyzed lipid concentrations in diets. (PDF)

S3 Table Serum and urine mineral concentrations. (PDF)

S4 Table Fecal excretion of total lipids and fatty acids during week 3 of the study. (PDF)

S5 Table Liver mRNA expression of lipogenic genes. (PDF)

S1 Fig Food consumption of rats. (PDF)
