# Supplementary material for: Dietary calcium affects body composition and lipid metabolism in rats
Source: PLoS One. 2019 Jan 10;14(1):e0210760. doi: 10.1371/journal.pone.0210760 (PMC6328234; doi:10.1371/journal.pone.0210760)
Supplement: S1 Table — (PDF) [file pone.0210760.s001.pdf]

S1 Table. Diet compositions.

Component	Diets				
	0.75Ca	2Ca	5Ca	10Ca	20Ca
Fixed ingredients (g/kg) [1]	709	709	709	709	709
Sucrose (g/kg)	288.227	285.105	277.612	265.125	240.15
Energy density (kcal/kg) [2]	4437 (18.57)	4425 (18.52)	4395 (18.40)	4345 (18.19)	4245 (17.77)
CHO (% of energy)	51.5	51.4	51.0	50.5	49.3
Lipid (% of energy)	32.9	33.0	33.2	33.6	34.4
Protein (% of energy)	15.6	15.6	15.7	15.9	16.3
Ca carbonate (g/kg)	1.873	4.995	12.488	24.975	49.95
Ca concentration (g/kg WW) [3]	0.789 ± 0.048^{e}	2.09 ± 0.01^{d}	5.08 ± 0.07^{c}	10.1 ± 0.1^{b}	20.1 ± 0.1^{a}

[1] Fixed ingredients (g/kg): cornstarch, 202.191; casein, 190; corn oil, 118; dyetrose, 75; anhydrous milkfat, 44.2; AIN-93G mineral mix without Ca (No.: 213019), 35; cellulose (microcrystalline), 30; AIN-93G vitamin mix (No.: 310025), 10; L-cystine, 3; choline bitartrate, 2.5; ethoxyquin, 0.009. [2] Energy density in MJ/kg is shown in parentheses. [3] Analyzed concentrations. Values are means ± SD, n = 5. Values in the row without a common superscript letter differ, $p < 0.05$. Ca: calcium; CHO: carbohydrate; WW: wet weight.
