# Supplementary material for: Dietary calcium affects body composition and lipid metabolism in rats
Source: PLoS One. 2019 Jan 10;14(1):e0210760. doi: 10.1371/journal.pone.0210760 (PMC6328234; doi:10.1371/journal.pone.0210760)
Supplement: S2 Table — (PDF) [file pone.0210760.s002.pdf]

S2 Table. Analyzed lipid concentrations in diets.

Lipid	Diets				
	0.75Ca (n = 6)	2Ca (n = 6)	5Ca (n = 6)	10Ca (n = 6)	20Ca (n = 6)
			g/kg WW		
10:0	0.465 ± 0.285	0.521 ± 0.253	0.442 ± 0.266	0.509 ± 0.235	0.429 ± 0.265
12:0	1.13 ± 0.09	1.20 ± 0.09	1.15 ± 0.08	1.21 ± 0.06	1.15 ± 0.10
14:0	4.52 ± 0.27	4.69 ± 0.16	4.57 ± 0.26	4.65 ± 0.07	4.63 ± 0.17
I15:0	0.081 ± 0.007	0.085 ± 0.003	0.081 ± 0.005	0.084 ± 0.003	0.084 ± 0.005
15:0	0.506 ± 0.033	0.514 ± 0.024	0.504 ± 0.029	0.512 ± 0.012	0.504 ± 0.027
I16:0	0.107 ± 0.010	0.109 ± 0.005	0.107 ± 0.007	0.109 ± 0.003	0.108 ± 0.005
16:0	27.8 ± 2.4	28.5 ± 1.5	27.8 ± 2.3	28.0 ± 0.7	28.2 ± 1.6
I17:0	0.151 ± 0.013	0.154 ± 0.010	0.152 ± 0.011	0.153 ± 0.005	0.152 ± 0.009
17:0	0.353 ± 0.031	0.365 ± 0.022	0.358 ± 0.031	0.359 ± 0.011	0.362 ± 0.025
18:0	6.79 ± 0.59	6.95 ± 0.42	6.82 ± 0.61	6.86 ± 0.18	6.91 ± 0.41
I18:0	0.024 ± 0.004	0.026 ± 0.004	0.026 ± 0.004	0.025 ± 0.002	0.026 ± 0.004
20:0	0.522 ± 0.048	0.532 ± 0.032	0.522 ± 0.048	0.523 ± 0.014	0.529 ± 0.031
22:0	0.187 ± 0.016	0.193 ± 0.014	0.188 ± 0.017	0.191 ± 0.006	0.192 ± 0.012
23:0	0.047 ± 0.005	0.047 ± 0.005	0.048 ± 0.005	0.047 ± 0.002	0.049 ± 0.005
24:0	0.210 ± 0.020	0.209 ± 0.019	0.209 ± 0.021	0.208 ± 0.007	0.211 ± 0.015
Total SFA	42.9 ± 3.2	44.1 ± 1.9	42.9 ± 3.0	43.4 ± 0.7	43.5 ± 2.0
16:1 9c	0.738 ± 0.067	0.758 ± 0.037	0.740 ± 0.064	0.747 ± 0.021	0.748 ± 0.039
16:1 11c	0.020 ± 0.001	0.023 ± 0.004	0.021 ± 0.003	0.021 ± 0.003	0.022 ± 0.003
16:1 13c	0.060 ± 0.006	0.064 ± 0.007	0.062 ± 0.006	0.063 ± 0.003	0.062 ± 0.006
17:1 9c	0.024 ± 0.007	0.028 ± 0.006	0.027 ± 0.004	0.026 ± 0.003	0.029 ± 0.007
18:1 9c	40.3 ± 3.7	41.1 ± 2.4	40.0 ± 3.7	40.2 ± 1.1	40.7 ± 2.5
18:1 11c	1.02 ± 0.09	1.04 ± 0.06	1.02 ± 0.09	1.02 ± 0.03	1.03 ± 0.06
18:1 12c	0.173 ± 0.019	0.188 ± 0.012	0.181 ± 0.018	0.182 ± 0.007	0.184 ± 0.010
18:1 13c	0.042 ± 0.006	0.047 ± 0.008	0.047 ± 0.004	0.044 ± 0.003	0.045 ± 0.003
18:1 14c	0.039 ± 0.004	0.039 ± 0.004	0.042 ± 0.008	0.038 ± 0.003	0.041 ± 0.002
18:1 15c	0.053 ± 0.007	0.056 ± 0.008	0.058 ± 0.005	0.053 ± 0.005	0.058 ± 0.004
Total 18:1 cis	41.6 ± 3.9	42.5 ± 2.4	41.4 ± 3.8	41.6 ± 1.2	42.1 ± 2.5
20:1 11c	0.360 ± 0.035	0.368 ± 0.021	0.358 ± 0.033	0.361 ± 0.010	0.364 ± 0.022
22:1 13c	0.026 ± 0.002[b]	0.028 ± 0.003[ab]	0.032 ± 0.002[a]	0.026 ± 0.002[b]	0.025 ± 0.002[b]
24:1 15c	0.016 ± 0.003	0.016 ± 0.005	0.015 ± 0.001	0.017 ± 0.001	0.015 ± 0.003
Total MUFA	42.9 ± 4.0	43.8 ± 2.5	42.6 ± 3.9	42.8 ± 1.2	43.4 ± 2.6
18:2 9c, 11t	0.125 ± 0.013	0.126 ± 0.006	0.126 ± 0.011	0.132 ± 0.004	0.128 ± 0.008

18:2 9t, 11t	0.098 ± 0.012	0.102 ± 0.010	0.100 ± 0.010	0.101 ± 0.007	0.102 ± 0.010
18:2 10t, 12c	0.018 ± 0.005	0.018 ± 0.003	0.019 ± 0.003	0.020 ± 0.003	0.020 ± 0.002
Total CLA	0.240 ± 0.028	0.247 ± 0.014	0.244 ± 0.021	0.253 ± 0.011	0.249 ± 0.017
18:2 n-6	66.6 ± 6.2	67.9 ± 3.9	65.9 ± 6.1	66.2 ± 1.9	67.1 ± 4.0
20:2 n-6	0.064 ± 0.007	0.067 ± 0.008	0.063 ± 0.007	0.066 ± 0.003	0.067 ± 0.007
20:3 n-6	0.060 ± 0.006	0.061 ± 0.004	0.060 ± 0.007	0.062 ± 0.002	0.060 ± 0.006
20:4 n-6	0.065 ± 0.007	0.067 ± 0.006	0.065 ± 0.008	0.065 ± 0.002	0.066 ± 0.006
22:4 n-6	0.021 ± 0.004	0.022 ± 0.003	0.021 ± 0.002	0.022 ± 0.003	0.019 ± 0.003
Total n-6 PUFA	66.8 ± 6.3	68.1 ± 3.9	66.1 ± 6.1	66.4 ± 1.9	67.3 ± 4.1
18:3 n-3	1.40 ± 0.13	1.43 ± 0.08	1.39 ± 0.13	1.40 ± 0.04	1.41 ± 0.09
22:5 n-3	0.042 ± 0.008	0.045 ± 0.008	0.040 ± 0.004	0.043 ± 0.006	0.044 ± 0.007
Total n-3 PUFA	1.44 ± 0.13	1.47 ± 0.09	1.43 ± 0.13	1.44 ± 0.05	1.46 ± 0.09
Total PUFA	68.3 ± 6.4	69.5 ± 4.0	67.6 ± 6.2	67.9 ± 2.0	68.8 ± 4.1
18:1 (6t-8t)	0.109 ± 0.010	0.108 ± 0.009	0.106 ± 0.011	0.107 ± 0.007	0.105 ± 0.008
18:1 9t	0.158 ± 0.012	0.164 ± 0.011	0.162 ± 0.013	0.161 ± 0.004	0.160 ± 0.007
18:1 10t	0.257 ± 0.020	0.268 ± 0.020	0.261 ± 0.022	0.266 ± 0.011	0.268 ± 0.014
18:1 11t	0.471 ± 0.037	0.480 ± 0.034	0.476 ± 0.042	0.478 ± 0.016	0.479 ± 0.034
18:1 12t	0.163 ± 0.012	0.168 ± 0.012	0.167 ± 0.015	0.168 ± 0.005	0.166 ± 0.009
18:1 (13t+14t)	0.385 ± 0.031	0.400 ± 0.027	0.395 ± 0.039	0.391 ± 0.010	0.394 ± 0.023
18:1 16t	0.154 ± 0.015	0.162 ± 0.011	0.161 ± 0.013	0.159 ± 0.005	0.159 ± 0.009
Total 18:1 TFA	1.70 ± 0.13	1.75 ± 0.11	1.73 ± 0.15	1.73 ± 0.05	1.73 ± 0.10
18:2 9t, 12t	0.108 ± 0.008	0.110 ± 0.007	0.109 ± 0.011	0.109 ± 0.008	0.109 ± 0.011
18:2 9c, 12t	0.328 ± 0.036	0.331 ± 0.022	0.320 ± 0.026	0.324 ± 0.012	0.328 ± 0.023
18:2 9t, 12c	0.224 ± 0.023	0.221 ± 0.016	0.222 ± 0.019	0.217 ± 0.014	0.221 ± 0.018
Total 18:2 TFA	0.660 ± 0.065	0.663 ± 0.044	0.651 ± 0.055	0.650 ± 0.031	0.658 ± 0.050
18:3 9t, 12c, 15c	0.106 ± 0.013	0.110 ± 0.006	0.106 ± 0.008	0.109 ± 0.005	0.109 ± 0.009
Total TFA	2.46 ± 0.21	2.52 ± 0.16	2.48 ± 0.21	2.49 ± 0.08	2.50 ± 0.16
Cholesterol [1]	0.178 ± 0.003	0.184 ± 0.005	0.181 ± 0.005	0.182 ± 0.002	0.181 ± 0.002
Total lipids	166 ± 5[ab]	170 ± 2[a]	163 ± 6[ab]	167 ± 3[a]	159 ± 4[b]

Values are means ± SD. Values in a row without a common superscript letter differ, $p < 0.05$. [1] n = 3. CLA: conjugated linoleic acid; MUFA: monounsaturated fatty acids; PUFA: polyunsaturated fatty acids; SFA: saturated fatty acids; TFA: trans fatty acids; WW: wet weight.
