# Supplementary material for: Dietary calcium affects body composition and lipid metabolism in rats
Source: PLoS One. 2019 Jan 10;14(1):e0210760. doi: 10.1371/journal.pone.0210760 (PMC6328234; doi:10.1371/journal.pone.0210760)
Supplement: S3 Table — (PDF) [file pone.0210760.s003.pdf]

S3 Table. Serum and urine mineral concentrations.

Parameter	Diet groups				
	0.75Ca	2Ca	5Ca	10Ca	20Ca
Serum Ca (mmol/L)	2.60 ± 0.07[c]	2.62 ± 0.11[bc]	2.67 ± 0.11[ac]	2.69 ± 0.11[ab]	2.70 ± 0.11[a]
Serum Mg (mmol/L)	0.84 ± 0.06[a]	0.76 ± 0.07[b]	0.74 ± 0.06[bc]	0.70 ± 0.05[c]	0.59 ± 0.08[d]
Serum P (mmol/L)	2.36 ± 0.24[a]	2.02 ± 0.19[c]	2.12 ± 0.18[bc]	2.20 ± 0.17[b]	2.42 ± 0.28[a]
Serum K (mEq/L)	4.65 ± 0.29[a]	4.63 ± 0.34[a]	4.49 ± 0.26[ab]	4.49 ± 0.30[ab]	4.40 ± 0.31[b]
Serum Na (mEq/L)	141 ± 1[ab]	140 ± 1[b]	141 ± 1[ab]	141 ± 1[a]	141 ± 1[ab]
Urine Ca (mg/g Cr)	11 ± 3[b]	12 ± 2[b]	12 ± 4[b]	16 ± 21[b]	82 ± 94[a]
Urine Mg (mg/g Cr)	93 ± 41	109 ± 33	93 ± 39	84 ± 36	108 ± 32
Urine P (mg/g Cr)	1315 ± 334[a]	1368 ± 206[a]	1072 ± 282[b]	817 ± 334[c]	287 ± 339[d]
Urine K (mg/g Cr)	2935 ± 685	2780 ± 391	2727 ± 576	2774 ± 519	2619 ± 623
Urine Na (mg/g Cr)	320 ± 188[a]	191 ± 97[b]	248 ± 153[ab]	195 ± 131[b]	224 ± 161[ab]

Values are means ± SD, n=27–30. Values in a row without a common superscript letter differ, $p < 0.05$. Ca: calcium; Cr: creatinine; K: potassium; Mg: magnesium; Na: sodium; P: phosphorus.
