# Supplementary material for: Dietary calcium affects body composition and lipid metabolism in rats
Source: PLoS One. 2019 Jan 10;14(1):e0210760. doi: 10.1371/journal.pone.0210760 (PMC6328234; doi:10.1371/journal.pone.0210760)
Supplement: S4 Table — (PDF) [file pone.0210760.s004.pdf]

S4 Table. Fecal excretion of total lipids and fatty acids during week 3 of the study.

Lipid	Diet groups				
	0.75Ca (n = 29)	2Ca (n = 28)	5Ca (n = 30)	10Ca (n = 30)	20Ca (n = 30)
	Fecal excretion (mg/wk)				
12:0	0.09 ± 0.03[c]	0.11 ± 0.08[c]	4.05 ± 1.78[b]	7.96 ± 3.11[a]	7.25 ± 3.36[a]
14:0	2.45 ± 0.48[c]	2.64 ± 0.67[c]	37.7 ± 14.6[b]	63.5 ± 22.3[a]	60.2 ± 23.0[a]
I15:0	1.03 ± 0.20[c]	0.97 ± 0.32[c]	1.56 ± 0.36[b]	2.27 ± 0.73[a]	2.45 ± 0.48[a]
15:0	3.78 ± 0.91[c]	4.02 ± 1.08[c]	13.5 ± 3.6[b]	19.0 ± 4.7[a]	18.5 ± 4.3[a]
I16:0	0.88 ± 0.22[c]	0.92 ± 0.33[c]	2.45 ± 0.71[b]	3.88 ± 1.35[a]	4.04 ± 1.13[a]
16:0	20.5 ± 4.9[c]	24.2 ± 8.8[c]	739 ± 275[b]	1160 ± 380[a]	1140 ± 380[a]
I17:0	0.38 ± 0.10[c]	0.43 ± 0.14[c]	3.32 ± 1.11[b]	5.50 ± 1.87[a]	5.57 ± 1.67[a]
17:0	0.88 ± 0.20[c]	0.91 ± 0.26[c]	12.1 ± 4.2[b]	18.0 ± 5.4[a]	18.0 ± 5.6[a]
18:0	9.88 ± 2.75[c]	12.4 ± 5.0[c]	330 ± 117[b]	431 ± 134[a]	433 ± 132[a]
I18:0	0.60 ± 0.16[c]	0.72 ± 0.36[c]	3.07 ± 0.93[b]	4.46 ± 1.53[ab]	4.78 ± 1.21[a]
20:0	1.82 ± 0.47[c]	2.23 ± 0.77[c]	28.8 ± 9.1[b]	38.7 ± 11.5[a]	39.8 ± 11.3[a]
22:0	2.72 ± 0.64[c]	2.98 ± 0.94[c]	13.5 ± 3.7[b]	17.6 ± 4.5[a]	18.4 ± 4.4[a]
23:0	1.14 ± 0.28[c]	1.21 ± 0.40[c]	3.64 ± 0.99[b]	4.50 ± 1.05[a]	4.65 ± 1.02[a]
24:0	5.05 ± 1.22[c]	5.76 ± 1.82[c]	22.7 ± 5.4[b]	28.6 ± 6.8[a]	30.8 ± 6.2[a]
Total SFA	51.2 ± 11.3[c]	59.5 ± 19.4[c]	1220 ± 430[b]	1800 ± 570[a]	1790 ± 570[a]
16:1 9c	0.50 ± 0.12[c]	0.45 ± 0.12[c]	1.49 ± 0.57[b]	4.17 ± 1.75[a]	4.08 ± 1.80[a]
16:1 11c	0.11 ± 0.03[d]	0.12 ± 0.03[d]	0.27 ± 0.09[c]	0.37 ± 0.12[b]	0.45 ± 0.13[a]
16:1 13c	1.15 ± 0.27[c]	1.06 ± 0.26[c]	1.79 ± 0.41[b]	2.45 ± 0.65[a]	2.55 ± 0.62[a]
17:1 9c	1.11 ± 0.34[c]	1.26 ± 0.33[c]	2.04 ± 0.34[b]	2.31 ± 0.55[a]	2.34 ± 0.38[a]
18:1 9c	8.07 ± 3.19[c]	8.71 ± 3.18[c]	148 ± 64[b]	490 ± 237[a]	494 ± 239[a]
18:1 11c	2.48 ± 0.49[c]	2.54 ± 0.69[c]	9.74 ± 3.50[b]	21.8 ± 8.8[a]	22.1 ± 8.5[a]
18:1 12c	0.12 ± 0.06[c]	0.13 ± 0.08[c]	2.53 ± 1.56[b]	3.10 ± 1.32[ab]	3.25 ± 1.17[a]
18:1 13c	0.15 ± 0.04[c]	0.15 ± 0.05[c]	1.03 ± 0.43[b]	1.45 ± 0.63[a]	1.58 ± 0.50[a]
18:1 14c	0.13 ± 0.02[c]	0.15 ± 0.05[c]	2.16 ± 0.69[b]	2.95 ± 0.98[a]	3.06 ± 0.87[a]
18:1 15c	0.04 ± 0.02[c]	0.05 ± 0.03[c]	1.21 ± 0.45[b]	1.69 ± 0.64[a]	1.76 ± 0.56[a]
Total 18:1 cis	11.0 ± 3.6[c]	11.7 ± 3.9[c]	165 ± 69[b]	521 ± 249[a]	526 ± 250[a]
20:1 11c	0.34 ± 0.11[c]	0.38 ± 0.14[c]	3.90 ± 1.72[b]	10.0 ± 4.2[a]	10.3 ± 4.3[a]
22:1 13c	0.30 ± 0.08[c]	0.29 ± 0.08[c]	1.94 ± 0.57[b]	3.02 ± 0.87[a]	3.42 ± 0.76[a]
24:1 15c	0.44 ± 0.11[d]	0.43 ± 0.13[d]	0.94 ± 0.23[c]	1.23 ± 0.33[b]	1.45 ± 0.36[a]
Total MUFA	14.9 ± 4.3[c]	15.7 ± 4.7[c]	177 ± 73[b]	545 ± 256[a]	551 ± 257[a]

18:2 9c, 11t	0.32 ± 0.22[c]	0.36 ± 0.19[c]	1.80 ± 1.35[b]	14.1 ± 14.9[a]	14.9 ± 18.7[a]
18:2 9t, 11t	0.44 ± 0.33[c]	0.42 ± 0.35[c]	1.73 ± 1.00[b]	10.6 ± 9.4[a]	11.9 ± 16.9[a]
18:2 10t, 12c	0.12 ± 0.07[c]	0.09 ± 0.05[c]	0.23 ± 0.08[b]	1.24 ± 1.23[a]	1.46 ± 2.39[a]
Total CLA	0.88 ± 0.57[c]	0.87 ± 0.56[c]	3.77 ± 2.37[b]	25.9 ± 24.8[a]	28.3 ± 37.4[a]
18:2 n-6	8.84 ± 3.53[c]	9.23 ± 3.48[c]	71.3 ± 27.2[b]	174 ± 76[a]	194 ± 108[a]
20:2 n-6	0.39 ± 0.08[c]	0.35 ± 0.10[c]	1.03 ± 0.33[b]	1.91 ± 0.72[a]	2.06 ± 0.83[a]
20:3 n-6	0.92 ± 0.40[c]	0.51 ± 0.30[c]	0.88 ± 0.45[b]	2.25 ± 1.05[a]	2.49 ± 1.11[a]
20:4 n-6	1.60 ± 0.63[c]	1.71 ± 0.98[c]	2.66 ± 1.01[b]	6.15 ± 2.64[a]	6.97 ± 2.72[a]
22:4 n-6	0.45 ± 0.11[d]	0.39 ± 0.12[d]	0.57 ± 0.16[c]	1.09 ± 0.36[b]	1.29 ± 0.39[a]
Total n-6 PUFA	12.4 ± 4.0[c]	12.4 ± 4.3[c]	76.9 ± 28.2[b]	186 ± 80[a]	209 ± 112[a]
18:3 n-3	1.60 ± 0.48[c]	1.73 ± 0.58[c]	8.74 ± 2.67[b]	18.6 ± 7.5[a]	20.2 ± 7.2[a]
22:5 n-3	0.11 ± 0.05[b]	0.12 ± 0.12[b]	0.15 ± 0.13[b]	0.46 ± 0.22[a]	0.56 ± 0.34[a]
Total n-3 PUFA	3.21 ± 0.88[c]	3.48 ± 1.06[c]	13.4 ± 3.5[b]	24.9 ± 9.4[a]	27.6 ± 8.8[a]
Total PUFA	15.6 ± 4.6[c]	15.9 ± 5.0[c]	90.3 ± 30.7[b]	211 ± 88[a]	236 ± 119[a]
18:1 (6t-8t)	0.08 ± 0.05[b]	0.11 ± 0.09[b]	3.61 ± 2.43[a]	2.86 ± 1.21[a]	2.96 ± 0.97[a]
18:1 9t	0.16 ± 0.06[c]	0.18 ± 0.08[c]	3.21 ± 1.51[b]	4.62 ± 1.74[a]	4.75 ± 1.64[a]
18:1 10t	0.54 ± 0.25[b]	0.62 ± 0.37[b]	6.13 ± 2.69[a]	7.69 ± 3.64[a]	8.06 ± 2.84[a]
18:1 11t	6.79 ± 3.57[c]	7.19 ± 3.64[c]	68.1 ± 46.9[b]	236 ± 117[a]	208 ± 96[a]
18:1 12t	0.22 ± 0.06[c]	0.25 ± 0.08[c]	2.90 ± 1.09[b]	4.91 ± 1.82[a]	4.99 ± 1.78[a]
18:1 (13t+14t)	0.29 ± 0.10[c]	0.35 ± 0.12[c]	9.75 ± 3.92[b]	15.2 ± 5.3[a]	15.1 ± 5.2[a]
18:1 16t	0.10 ± 0.03[c]	0.12 ± 0.05[c]	3.97 ± 1.36[b]	6.12 ± 2.00[a]	6.22 ± 2.04[a]
Total 18:1 TFA	8.18 ± 3.96[c]	8.81 ± 4.14[c]	97.6 ± 57.0[b]	278 ± 131[a]	250 ± 106[a]
18:2 9c, 12t	0.09 ± 0.05[c]	0.10 ± 0.04[c]	1.37 ± 0.49[b]	3.43 ± 1.46[a]	3.44 ± 1.60[a]
18:2 9t, 12c	0.90 ± 0.30[c]	1.16 ± 0.51[c]	4.66 ± 1.42[b]	6.78 ± 2.71[a]	7.78 ± 2.43[a]
Total 18:2 TFA	0.99 ± 0.33[c]	1.25 ± 0.54[c]	6.03 ± 1.77[b]	10.2 ± 4.0[a]	11.2 ± 3.6[a]
18:3 9t, 12c, 15c	0.05 ± 0.02[c]	0.06 ± 0.03[c]	1.07 ± 0.53[b]	2.83 ± 1.18[a]	2.87 ± 1.21[a]
Total TFA	9.23 ± 4.17[c]	10.1 ± 4.5[c]	105 ± 59[b]	291 ± 135[a]	264 ± 110[a]
Total lipids (g/wk)	0.62 ± 0.11[c]	0.67 ± 0.20[c]	2.18 ± 0.70[b]	3.57 ± 1.13[a]	3.42 ± 1.08[a]

Values are means ± SD. Values in a row without a common superscript letter differ, $p < 0.05$. CLA: conjugated linoleic acid; MUFA: monounsaturated fatty acids; PUFA: polyunsaturated fatty acids; SFA: saturated fatty acids; TFA: trans fatty acids; wk: week.
