# Supplementary material for: Dietary calcium affects body composition and lipid metabolism in rats
Source: PLoS One. 2019 Jan 10;14(1):e0210760. doi: 10.1371/journal.pone.0210760 (PMC6328234; doi:10.1371/journal.pone.0210760)
Supplement: S5 Table — (PDF) [file pone.0210760.s005.pdf]

S5 Table. Liver mRNA expression of lipogenic genes.

Gene	Diet groups				
	0.75Ca (n = 29)	2Ca (n = 28)	5Ca (n = 30)	10Ca (n = 30)	20Ca (n = 30)
Cyp7a1	1.23 ± 1.37	0.89 ± 0.66	1.00 ± 0.94	1.35 ± 1.71	1.95 ± 2.07
Ldlr	0.73 ± 0.39[d]	0.70 ± 0.31[d]	1.00 ± 0.50[c]	1.42 ± 0.58[b]	2.64 ± 1.28[a]
Hmgcr	0.55 ± 0.28[d]	0.74 ± 0.42[cd]	1.00 ± 0.58[c]	1.71 ± 1.23[b]	2.51 ± 1.20[a]
Hmgcs1	0.63 ± 0.37[d]	0.75 ± 0.40[cd]	1.00 ± 0.53[c]	1.53 ± 0.66[b]	3.40 ± 1.80[a]
Fasn	0.27 ± 0.16[c]	0.46 ± 0.42[b]	1.00 ± 1.29[a]	0.93 ± 0.68[a]	0.87 ± 0.61[a]
Cpt2	0.37 ± 0.19[e]	0.59 ± 0.33[d]	1.00 ± 0.39[c]	1.61 ± 0.52[b]	2.52 ± 0.91[a]
Acat2	0.61 ± 0.27[d]	0.65 ± 0.31[d]	1.00 ± 0.54[c]	1.69 ± 0.72[b]	3.71 ± 1.94[a]

Relative mRNA expressions of lipid metabolism genes in liver (normalized to 18S rRNA). Results are presented as means ± SD. The mean of the 5Ca group (normal calcium) was arbitrarily set to 1. Values in a row without a common superscript letter differ, $p < 0.05$. *Acat2*: acetyl-CoA acetyltransferase 2; *Cpt2*: carnitine palmitoyltransferase 2; *Cyp7a1*: cholesterol 7 alpha-hydroxylase; *Fasn*: fatty acid synthase; *Hmgcr*: 3-hydroxy-3-methylglutaryl-CoA reductase; *Hmgcs1*: 3-hydroxy-3-methylglutaryl-CoA synthase 1; *Ldlr*: low-density lipoprotein receptor.
