# Supplementary material for: Dietary calcium affects body composition and lipid metabolism in rats
Source: PLoS One. 2019 Jan 10;14(1):e0210760. doi: 10.1371/journal.pone.0210760 (PMC6328234; doi:10.1371/journal.pone.0210760)
Supplement: S1 Fig — (PDF) [file pone.0210760.s006.pdf]

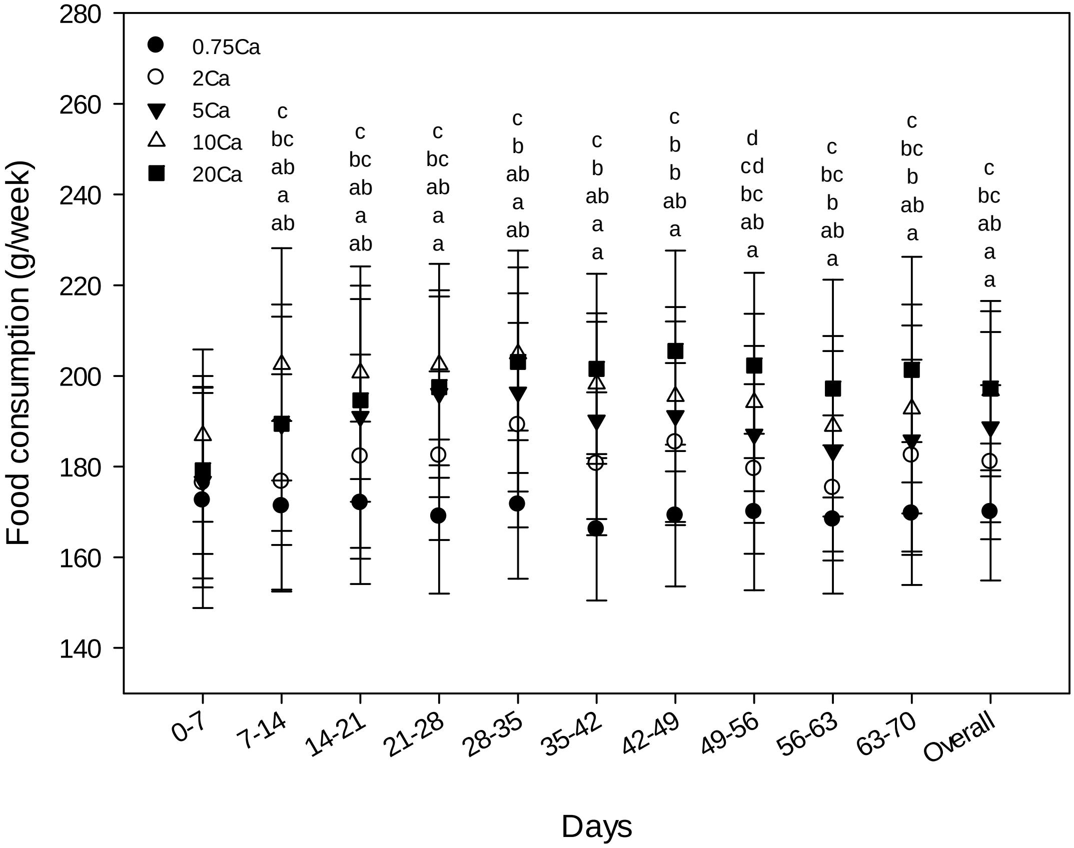

S1 Fig. Food consumption of rats. Results are presented as means ± SD, n = 28–30. Results were analyzed by mixed-design ANOVA to determine effects and interaction of diet and time. For time points with a significant ($p < 0.05$) diet effect, differences among diet groups were determined using one-way ANOVA followed by the Holm-Sidak post-hoc test. Diet groups without a common letter differ, $p < 0.05$. Letters correspond to diet groups 0.75Ca, 2Ca, 5Ca, 10Ca and 20Ca sequentially from top to bottom. Overall food consumption was calculated by dividing the total food consumption for the entire study by the number of weeks of the study (10 weeks).
